# Adherence with Dosing Guideline in Patients with Impaired Renal Function at Hospital Discharge

**DOI:** 10.1371/journal.pone.0128237

**Published:** 2015-06-08

**Authors:** A. Clara Drenth-van Maanen, Rob J. van Marum, Paul A. F. Jansen, Jeannette E. F. Zwart, Wouter W. van Solinge, Toine C. G. Egberts

**Affiliations:** 1 University Medical Center Utrecht, Department of Geriatrics, Utrecht, The Netherlands; 2 Expertise Centre Pharmacotherapy for Old persons (Ephor), Utrecht, The Netherlands; 3 Jeroen Bosch Hospital, Department of Geriatrics, ‘s-Hertogenbosch, The Netherlands; 4 VUmc, Department of General Practice & Elderly Care Medicine, EMGO+ Institute for Health and Care Research, Amsterdam, The Netherlands; 5 University Medical Center Utrecht, Department of Clinical Pharmacy, Utrecht, The Netherlands; 6 University Medical Center Utrecht, Department of Clinical Chemistry and Haematology, Utrecht, The Netherlands; 7 Utrecht Institute for Pharmaceutical Sciences (UIPS), Department of Pharmacoepidemiology and Pharmacotherapy, Faculty of Science, Utrecht University, Utrecht, the Netherlands; University of Perugia, ITALY

## Abstract

**Objectives:**

To determine the prevalence, determinants, and potential clinical relevance of adherence with the Dutch dosing guideline in patients with impaired renal function at hospital discharge.

**Design:**

Retrospective cohort study between January 2007 and July 2011.

**Setting:**

Academic teaching hospital in the Netherlands.

**Subjects:**

Patients with an estimated glomerular filtration rate (eGFR) between 10-50 ml/min/1.73m^2^ at discharge and prescribed one or more medicines of which the dose is renal function dependent.

**Main Outcome Measures:**

The prevalence of adherence with the Dutch renal dosing guideline was investigated, and the influence of possible determinants, such as reporting the eGFR and severity of renal impairment (severe: eGFR<30 and moderate: eGFR 30-50 ml/min/1.73m^2^). Furthermore, the potential clinical relevance of non-adherence was assessed.

**Results:**

1327 patients were included, mean age 67 years, mean eGFR 38 ml/min/1.73m^2^. Adherence with the guideline was present in 53.9% (n=715) of patients. Reporting the eGFR, which was incorporated since April 2009, resulted in more adherence with the guideline: 50.7% vs. 57.0%, RR 1.12 (95% CI 1.02-1.25). Adherence was less in patients with severe renal impairment (46.0%), compared to patients with moderate renal impairment (58.1%, RR 0.79; 95% CI 0.70-0.89). 71.4% of the cases of non-adherence had the potential to cause moderate to severe harm.

**Conclusion:**

Required dosage adjustments in case of impaired renal function are often not performed at hospital discharge, which may cause harm to the majority of patients. Reporting the eGFR can be a small and simple first step to improve adherence with dosing guidelines.

## Introduction

A reduction in glomerular filtration rate (GFR) decreases the elimination rate of medications or their metabolites that are primarily excreted by the kidneys. In that case, these medications can accumulate if prescribed in the normal, standard dosage, which may lead to exaggerated pharmacologic effects or adverse drug reactions. As such, dosage reduction is required in patients with impaired renal function.

Several studies have demonstrated that during hospital stay dosages in patients with impaired renal function are not adjusted in 25–77% of cases;[1–5] 63% of these prescriptions potentially have adverse consequences, and 3% have been rated as having a potential for fatal or severe consequences.[6] Inappropriately high dosage prescribing at discharge occurs in 25–88% of prescriptions.[1, 3, 4, 6–10]

A possible explanation for these observations is that impaired renal function is insufficiently acknowledged. In 42–57% of patients impaired renal function is not mentioned in the medical chart.[7, 11, 12] Furthermore, if only serum creatinine levels are used to estimate renal function, particularly older patients are susceptible for insufficient acknowledgment, since serum creatinine is an inaccurate measure of GFR, particularly in frail and/or malnourished older persons.[13] Equations to estimate GFR, such as the Modification of Diet in Renal Disease (MDRD), predict GFR better than serum creatinine levels.[14] Therefore, clinical laboratories increasingly report estimated (e)GFR values whenever the serum creatinine level is measured. The abovementioned studies on the prevalence of inappropriate dosage prescribing all seem to have been performed before the introduction of standard reporting the eGFR in addition to the serum creatinine levels in laboratory results. Furthermore, these studies focused on one specific medication group, such as antibiotics, or one specific patient population (such as patients admitted to the internal ward).

The aim of the current investigation was to analyse the prevalence and determinants of adherence with the Dutch dosing guideline in patients with impaired renal function at hospital discharge, thereby including multiple patient populations, multiple medication groups, and the influence of reporting the eGFR in addition to serum creatinine levels. In addition, the potential clinical relevance of non-adherence was assessed.

## Methods

### Design and setting

A retrospective cohort study was conducted at the University Medical Center Utrecht (UMCU), a 1042-bed academic teaching hospital in the Netherlands. In the UMCU, all medications for hospitalised patients are prescribed using a computerised physician order entry (CPOE) system. All prescriptions are routinely exported to the Utrecht Patient Oriented Database (UPOD). UPOD is an infrastructure of relational databases comprising data on patient demographics, hospital discharge diagnoses, medical procedures, medication orders and laboratory tests for all patients treated at the UMCU since 2004, and has been described in detail elsewhere.[15] The establishment and utilization of UPOD is in accordance with guidance of the Institutional Review Board (IRB) and privacy board of UMC Utrecht, which allows the use of clinical data from patients who did not object to use of their data for scientific purposes, as long as the patients cannot be identified directly from the data. Within UPOD, only data are captured that were initially registered during routine care and not for research purposes. Because no extra material, for example, blood samples, is taken from patients, there is not a requirement to obtain informed consent from individual patients or seek IRB approval for every study protocol. Patients are informed at the time of admission that their data can be used for scientific research purposes. Patients can object to the use of their data within UPOD according to a general procedure for objecting to the use of data for scientific research that is available at UMC Utrecht.

### Study population

All patients, aged ≥ 18 years, with an estimated (e)GFR between 10–50 ml/min/1.73m^2^, based on the last measured serum creatinine level during hospital admission, discharged between January 2007 and July 2011, and using at least one of the defined set of 41 medications (see Outcome) were eligible for inclusion. Exclusion criteria were unknown renal function, patients undergoing dialysis, death during hospitalisation, no medications at discharge, and discharge within 24 hours after admission. Patients with an eGFR below 10 ml/min/1.73m^2^ were not included; since they usually are on dialysis and dosing guidelines generally do not offer standard dosage advice in these cases. Only the first admission of patients during the study period was included.

GFR was estimated by the three-variable version of the MDRD-formula:[16] eGFR (ml/min./1.73m2) = 175 x (serum creatinine (μmol/l)/88.4)– 1.154 x (age in years)– 0.203 (x 0.74 if female).

### Outcome

The primary outcome was the prevalence of adherence with the Dutch dosing guideline, the G-standard, in the last 24 hours before discharge.

The Dutch dosing guideline is an evidence based and professional guideline for drug dosing in renal failure, developed and maintained by a multidisciplinary working group of the Scientific Institute for Dutch Pharmacists. For the period 2007–2009 the guideline 2007 was applied,[17] for the period 2009–2011 the guideline 2009 was applied.[18] In the guideline, dosing advices are provided for each drug according to renal impairment category. Renal impairment is categorized according to the European Medicines Agency (EMA) guideline: moderate: eGFR 30–50 ml/min/1.73m^2^ and severe: eGFR 10–29 ml/min/1.73m^2^ renal impairment.[19]

When the guideline offered multiple dosage advices, e.g. in case of multiple possible indications, the highest dosage advice was applied, as drug indication could not be retrieved from the database. This prevents an underestimation of adherence. Medications that did not require dosage adjustment in one of the indications were not evaluated, since in that case adherence with the guideline could not be assessed. Furthermore, medications requiring dosage adjustments based on plasma levels, body surface/weight, and monitoring of therapeutic effect were not evaluated, since this information could not be retrieved from the UPOD database.

This left 41 medications for which the prescribed dosage could be compared to the advised maximum daily dose in the Dutch dosing guideline. These medications are listed and categorized according to the Anatomical Therapeutic Chemical (ATC) Classification System classes in Table 1.

**Table 1:** List of the 41 analysed medications.

Medication	
Cardiovascular system	
	acebutolol
	sotalol
	rosuvastatin
	atenolol
	hydrochlorothiazide
	eplerenone
	bisoprolol
	chlorthalidone
	amiloride
	pentoxifylline
	epitizide/triamtereen
	acipimox
	indapamide
Musculo-skeletal system	
	allopurinol
	colchicine
	alendroninic acid
	benzbromarone
	risedronic acid
	clodronic acid
	etidronic acid
	sodiumaurothiomalate
Alimentary tract and metabolism	
	metoclopramide
	cimetidine
	ranitidine
	metformine
	famotidine
Nervous system	
	tramadol
	gabapentin
	hydroxyzine
	pregabalin
	levetiracetam
	chloralydrate
	amantadine
Antiinfectives for systemic use	
	clavulanic acid
	valganciclovir
	nitrofurantoin
	norfloxacin
	clarithromycin
	levofloxacin
	aciclovir
Respiratory system	
	levocetirizine
	cetirizine
Genito urinary system	
	solifenacin
	tolterodine

### Determinants

The influence of the following potential determinants on adherence with the dosing guideline were studied:

- age (18–64 years, 65–79 years, 80+ years)
- gender
- reporting of serum creatinine levels only vs. additional reporting of eGFR (in the UMCU this was incorporated since April 2009)
- renal impairment category (last measured eGFR before discharge, severe: 10–29 ml/min/1.73m^2^, moderate: 30–50 ml/min/1.73m^2^)
- change in renal impairment category during hospital admission (stable, i.e. no change in renal impairment category, declining, i.e. decrease in renal function by one or more renal impairment categories, or improving, i.e. increase in renal function by one or more renal impairment categories)
- length of hospital stay in days (≤ 7 days, 8–30 days, or > 30 days)
- admitting medical specialty (surgical vs. non-surgical)

The secondary outcome measure was the potential clinical relevance of non adherence with the dosing guideline. This was determined by two clinical geriatricians-clinical pharmacologists (RM and PJ) according to the classification system as described by Cornish et al.[20] This system consists of three classes, class 1 (unlikely to cause harm); class 2 (potential to cause moderate discomfort or clinical deterioration, such as nausea or diarrhea); and class 3 (potential to cause severe discomfort or clinical deterioration, such as extension of hospital stay).

### Data analysis

Descriptive statistics were used to describe the following characteristics at baseline: age, gender, renal impairment category, length of hospital stay, admitting medical specialty, and number of prescriptions per patients that required dosage adjustment.

The prevalence of adherence per renal impairment category (moderate and severe) was calculated by dividing the number of adjusted dosage prescriptions by the total number of prescriptions that required dosage adjustment. Also, the prevalence rates of adherence with the Dutch professional guideline were calculated per evaluated medication in the two renal impairment categories.

The prevalence of patients receiving dosage prescriptions in accordance with the Dutch dosing guideline was determined through dividing the number of patients receiving only prescriptions with dosages adjusted according to the dosing guideline by the total number of included patients. In addition, these results were stratified to age (18–65, 66–80, and >80 years), gender, reporting of eGFR (yes or no), renal impairment category (moderate or severe), change in renal impairment category (stable, declining, or improving), length of hospital stay (≤ 7 days, 8–30 days, or > 30 days), and admitting medical specialty (surgical or non-surgical). The results of stratification were expressed as relative risks (RR) with 95% confidence intervals.

## Results

**Fig 1:**
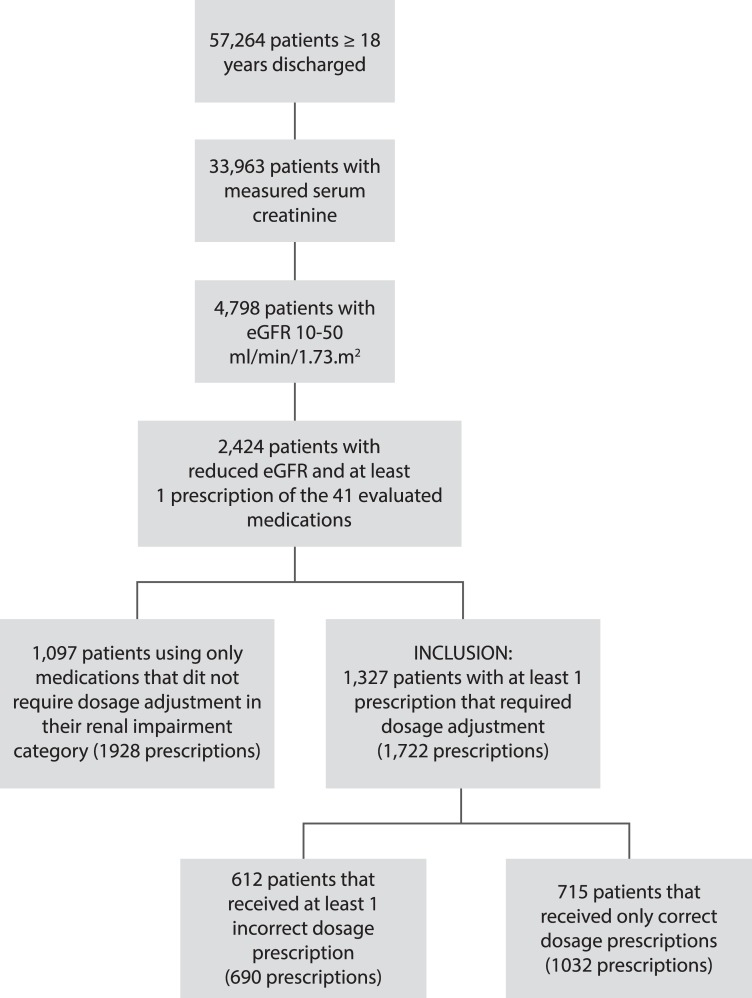
Flowchart of inclusion.

During the study period 57,264 patients ≥ 18 years were discharged from the UMCU. In 33,963 (59.3%) patients a serum creatinine level was measured during hospital admission. Of those patients, 4798 (14.1%) had an eGFR between 10 and 50 ml/min/1.73m^2^. Eventually, 1327 patients met all inclusion criteria and their data were used for further analysis (Fig 1; S1 Table).

Mean age of the included patients was 67 years (range 18–99), 50.2% was male, and mean length of hospital stay was 12 days (Table 2). Severe renal impairment (eGFR 10–29 ml/min/1.73m^2^) was present in 35.5% of these patients. The remaining 64.5% of patients had moderate renal impairment (eGFR 30–50 ml/min/1.73m^2^).

**Table 2:** Baseline characteristics.

Characteristics	n = 1327
Age in years, mean (range)	67 (18–99)
Male gender, n (%)	668 (50.3)
Median length of hospital admission in days, n (range)	7 (1–261)
Admitted to surgical medical specialty, n (%)	443 (33.4)
MDRD at discharge 10–29 ml/min/1.73m2, n (%)	461 (34.7)
MDRD at discharge 30–50 ml/min/1.73m2, n (%)	866 (65.3)
Median number of prescriptions requiring dosage adjustment per patient, n (range)	1 (1–4)

### Prevalence of adherence with the Dutch dosing guideline

The 1327 patients received 1722 prescriptions in which dosage adjustment was required according to renal impairment category. Adherence with the Dutch dosing guideline was present in 53.9% (n = 715) of the patients and in 59.9% (n = 1032) of the prescriptions. In the cases of non-adherence 26.8% (n = 185), concerning 13 different medications, were actually contra-indicated. On average, medications were prescribed in twice the maximum advised daily dose, range 1.25–8. Five of the investigated medications were in all cases prescribed according to maximum advised daily dose: acipimox, bisoprolol, cimetidine, clarithromycin, and hydroxyzine.

Cardiovascular medications were most frequently prescribed (Table 3): 27.9% (n = 481) of the prescriptions requiring dosage adjustment, followed by medications from the ATC-classes musculoskeletal system and alimentary tract and metabolism (21.8%, n = 376, and 18.6%, n = 320, of the prescriptions, respectively). Within the cardiovascular group, dosages were adjusted in 67.2% (n = 323) of the prescriptions, cases of non-adherence mostly concerned eplerenone (n = 54). In the musculoskeletal system and alimentary tract and metabolism groups, dosages were adjusted in 58.5% en 47.8% of the prescriptions, respectively, cases of non-adherence mainly concerned colchicine, allopurinol, and metoclopramide. Relatively least adherence with the Dutch dosing guideline was found in medications acting on the respiratory system (2.4%: levocetirizine and cetirizine), genito urinary system (33.3%: tolterodine and solifenacin), and anti-infectives for systematic use (39.4%: clavulanic acid, valganciclovir, nitrofurantoin, norfloxacin, clarithromycin, levofloxacin, and aciclovir).

**Table 3:** Adherence with the Dutch dosing guideline.

	MDRD 30–50 ml/min/1.73m^2^			MDRD 10–29 ml/min/1.73m^2^		
Medication	Prescriptions (n)	adherent (n)	%	Prescriptions (n)	adherent (n)	%
**Cardiovascular system**	323	249	77.1	158	74	46.8
eplerenone**	48	0	0	6	0	0
sotalol	156	136	87.2	26	12	46.2
hydrochlorothiazide*	--------------	------------	------	34	0	0
atenolol	--------------	------------	------	38	26	68.4
rosuvastatin	102	102	100	14	12	85.7
bisoprolol	--------------	------------	------	24	24	100
other	17	11	64.7	16	0	0
**Musculo-skeletal system**	234	145	62.0	142	75	52.8
colchicine	91	38	41.8	47	26	55.3
allopurinol	138	104	75.4	65	49	75.4
alendroninic acid*	--------------	------------	------	14	0	0
benzbromarone*	--------------	------------	------	15	0	0
other	5	3	60.0	1	0	0
**Al. tract and metabol.**	228	120	52.6	92	33	35.9
metoclopramide	228	120	52.6	51	32	62.7
metformine*	--------------	------------	------	28	0	0
ranitidine	--------------	------------	------	11	9	80.9
other	--------------	------------	------	2	1	50
**Nervous system**	165	147	89.1	134	123	91.8
tramadol	--------------	------------	------	91	86	94.5
gabapentin	36	31	86.1	9	7	77.8
pregabalin	55	51	92.7	14	12	85.7
levetiracetam	36	31	86.1	9	7	77.8
hydroxyzine	34	34	100	11	11	100
other	4	0	0	0	n.a.	n.a.
**Antiinf. for syst. use**	70	26	37.1	85	35	41.2
clavulanic acid	--------------	------------	------	44	10	22.7
valganciclovir	48	24	50.0	36	23	63.9
nitrofurantoin**	19	0	0	2	0	0
other	3	2	66.7	3	2	66.7
**Respiratory system**	57	1	1.8	25	1	4.0
levocetirizine	40	0	0	13	0	0
cetirizine	17	1	5.9	12	1	8.3
**Genito urinary system**	--------------	------------	------	9	3	33.3
solifenacin	--------------	------------	------	6	3	50.0
tolterodine	--------------	------------	------	3	0	0
**Total**	**1077**	**688**	**63.9**	**645**	**344**	**53.3**

---- = no dosage adjustment required

n.a. = not applicable

* = contra-indicated drug in case of MDRD < 30 ml/min/1.73m^2^

** = contra-indicated drug in case of MDRD ≤ 50 ml/min/1.73m^2^

Al. tract and metabol. = alimentary tract and metabolism

Antiinf. for syst. use = antiinfectives for systemic use

### Determinants of adherence with the dosing guideline

Adherence with the Dutch dosing guideline was not influenced by age, gender, or length of hospital stay (Table 4). Reporting the eGFR in the laboratory values in addition to serum creatinine levels only, was associated with more adherence with the dosing guideline: 50.7% adherence if only creatinine levels were reported vs. 57.0% if also eGFR was reported, RR 1.12 (95% CI 1.02–1.25). Adherence with the dosing guideline was less prevalent in patients with severe renal impairment (46.0%), compared to patients with moderate renal impairment (58.1%): RR 0.79 (95% CI 0.70–0.89). Also, an improving renal function during hospital admission was stronger associated with adherence with the dosing guideline (72.5%), and less associated in case of a declining renal function (44.9%), compared to cases with stable renal function: RR1.39 (95% CI 1.24–1.54) vs. RR 0.86 (95% CI 0.73–1.01). Furthermore, adherence was better in patients discharged from a surgical department (58.9%), compared with patients discharged from a non-surgical department (51.4%): RR 1.15 (95% CI 1.03–1.27).

**Table 4:** Determinants of adherence with dosing guideline.

Characteristic	N (patients)	N patients with all dosages adjusted according to the guideline	RR (95% CI)
**Total**	1327	715 (53.9%)	
**Age, y**			
18–64	513	264 (51.5%)	ref
65–79	572	324 (56.6%)	1.10 (0.98–1.23)
≥80	242	127 (52.5%)	1.02 (0.87–1.18)
**Gender**			
Male	668	372 (55.7%)	ref
Female	659	343 (52.0%)	0.94 (0.84–1.04)
**Reporting the eGFR**			
No	655	332 (50.7%)	ref
Yes	672	383 (57.0%)	1.12 (1.02–1.25)
**MDRD, ml/min/1.73m^2^**			
30–50	866	503 (58.1%)	ref
10–29	461	212 (46.0%)	0.79 (0.70–0.89)
**Change in renal impairment during hospital admission**			
Stable	893	465 (52.1%)	ref
Declining 1 or more categories	234	105 (44.9%)	0.86 (0.73–1.01)
Improving 1 or more categories	200	145 (72.5%)	1.39 (1.24–1.54)
**Duration of stay, days**			
≤ 7 days	585	308 (52.6%)	0.95 (0.85–1.06)
8–30 days	653	362 (55.4%)	ref
≥ 31 days	89	45 (50.6%)	0.91 (0.71–1.12)
**Prescriber**			
Nonsurgical	884	454 (51.4%)	ref
Surgical	443	261 (58.9%)	1.15 (1.03–1.27)

As CKD is not documented in the UPOD, a subanalysis was conducted in patients admitted to the renal department, as in this population the proportion of patients with CKD is probably high. During the study period 334 patients were admitted to the renal department, 74% of patients left the hospital without dosing errors in their prescribed medications.

### Potential clinical relevance of non-adherence with the dosing guideline

All cases of non-adherence had the potential to cause harm, of which 71.4% had the potential to cause moderate to severe harm. An example is the prescription of eplerenone, which is contra-indicated in cases of moderate to severe renal impairment due to the risk of increased serum potassium values, which may cause arrhythmia.

In 50 patients we checked the actual discharge medication list for dosage modifications compared to the medication list prescribed in the last 24 hours before discharge. These patients were prescribed 78 medications without the necessary dosage adjustments. Ten (12.8%) of these medications were modified at discharge; in five cases highly dosed antibiotics were discontinued at discharge because the antibiotic therapy had ended; in one case an highly dosed antibiotic was stopped due to an allergic reaction; in two cases all medications were discontinued because of terminal disease; and in two cases (2.6%) medication dosages were intentionally adjusted to renal function at discharge.

## Discussion

The results of the present study indicate that adherence with the Dutch dosing guideline at hospital discharge in patients with renal impairment is only 53.9%.

In this study, adherence with the dosing guideline was investigated in multiple medication groups in a large study population. Furthermore, multiple potential determinants were assessed on their influence on the prevalence of non-adherence with the dosing guideline. Another important aspect of this study is that the potential clinical relevance of non-adherence was determined. In the majority of cases, they had the potential to cause moderate to severe discomfort or clinical deterioration, which may lead to (extension of) hospital stay.

Reporting the eGFR was associated with more adherence with the dosing guideline. Non adherence was most frequently found in patients with severe renal impairment. Most cases of non-adherence were identified in cardiovascular medications, especially eplerenone, drugs acting on the musculoskeletal system, especially allopurinol and colchicine, and drugs acting on the alimentary tract and metabolism, especially metoclopramide. Furthermore, in none of the cases the prescribers were adherent with the maximum advised daily dose for levocetirizine.

Physicians from surgical specialties were more adherent with the dosing guideline than physicians from non-surgical specialties. A possible explanation for this observation is that in the UMCU surgeons routinely consult a nephrologist when they treat patients with impaired renal function. Another explanation is that in general surgeons prescribe less new medications during hospital admission.

As expected, best adherence was found in patients admitted to the nephrology ward. In this population we assume that a significant part of patients has known kidney disease, which may lead to an increased alertness to adjust drug dosages to renal function. This in combination with more knowledge of which medications need dosage adjustment in case of reduced renal function, explains why adherence was best in patients admitted to the nephrology ward.

The prevalence rates of adherence with the dosing guideline found in this study were comparable to prevalence rates reported in other studies.[1, 9] In order to improve adherence with dosing guidelines, physicians first need to identify patients with impaired renal function, and second to identify medication that require dosage adjustment. Reporting the eGFR helps to identify patients with impaired renal function. Previous studies have shown conflicting results for the effect of reporting the eGFR on adherence with dosing guidelines.[21, 22] Although the present study showed that adherence with the dosing guideline improved significantly after introduction of reporting the eGFR, the absolute effect was relatively small. Routinely consulting a nephrologist will probably be more effective, as the results of this study showed that surgeons, who routinely consult a nephrologists, adhere better with the dosing guideline than other physicians. However, nephrologists do not have the capacity to be involved with all hospitalized patients with impaired renal function. Therefore, dose adjust alerts for drug dosage adjustment by computerized clinical decision support systems (CDSS) may be the most feasible and effective intervention. Dose adjust alerts assist physicians with identifying medications that require dosage adjustment. Various studies have investigated the effect of dose adjust alerts, and except for the study of Sellier et al., these studies confirmed that dose adjust alerts improve adherence with dosing guidelines. [23–26] The study of Sellier et al. had a low baseline inappropriate dosage prescribing rate of 21%, probably because of pharmacists already reviewed all prescriptions. This may implicate a ceiling effect, and therefore explain the limited impact of dose adjust alerts in this study. Consequently, hospitals with a high baseline non adherence rate can benefit from dose adjust alerts. Also in primary care dose adjust alerts have proven to be effective.[27] Reporting the eGFR, which is currently done by more than 80% of clinical laboratories in the USA, could be a simple first step to improve adherence with dosing guidelines.[28]

The present study was subject to several limitations. First, it was conducted in only one hospital, which limits the generalizability of the study results. Second, GFR was estimated by the three-variable version of the MDRD formula, which did not correct for race and body surface area. For patients at the extremes of muscle mass or those with an unusual diet or a condition associated with changes in creatinine secretion, the MDRD estimate, which is based on serum creatinine, is likely to be inaccurate.[29] This is particularly relevant for populations who are most likely to require medications, such as the frail older patients, critically ill, or cancer patients. This limitation holds for all serum creatinine-based formulas, although the Cockcroft-Gault formula seems to perform better in those populations, as well as cystatin C based formulas.[30, 31] Third, the actual discharge prescriptions could not be assessed due to limitations of the database. Therefore, the prescriptions in the last 24 hours before discharge were assessed. These could have differed from the actual discharge prescriptions. At the time of providing the discharge prescriptions, physicians may have performed a last medication check and adjust dosage prescriptions. However, in a small subpopulation we assessed the actual discharge medication list and found that in only 2.6% of cases medication was indeed intentionally adjusted to renal function at discharge. Fourth, as we had no information on pre-admission medication use, we could not assess whether prescriptions were initiated in the hospital or before hospital admission. Fifth, since this was a large retrospective cohort study, we could not study dosages in individual cases, for example on indication. Therefore we chose to set the maximum possible dose in each medication as high as possible. This implicates that it is possible that we have made an overestimation of adherence with the dosing guideline. Sixth, intentional non adherence with the dosing guideline could not be assessed. Seventh, we were not able to correct the eGFR for race. In the UMC Utrecht, race is not routinely registered; therefore we had no data on race in our study population. We tried to estimate the percentage of black people in the Netherlands, however this is also not documented. We consulted ‘Statistics Netherlands’, a Dutch organization that is responsible for collecting and processes data in order to publish statistics to be used in practice, by policymakers and for scientific research, but they have no data on the percentage of people from the black race in the Netherlands.[32] Finally, we relied on the Dutch dosing guideline. This guideline may vary from other internationally used dosing guidelines. Though, because the guideline is based on international research results, we do not expect that these variations have major clinical implications.

## Conclusion

In patients with impaired renal function medication dosages are often not adjusted at hospital discharge. Especially in patients with severe renal impairment and/or declining renal function required dosage adjustments are frequently not performed. Non adherence with the dosing guideline was mainly found for eplerenone, allopurinol, colchicine, metoclopramide, and levocetirizine and was potentially harmful in the majority of cases. Although reporting the eGFR improves adherence with dosing advices, the prevalence of non-adherence remains high. Nonetheless, reporting the eGFR may be a simple first step to improve adherence with dosing guidelines. Implementations of dosing guidelines into CPOE systems will probably further improve prescribing behavior of physicians.

## Supporting Information

S1 Table UPOD database. (XLSX)
